# Negative Appendicectomy Rates in Females of Childbearing Age: A Retrospective Analysis and Literature Review

**DOI:** 10.7759/cureus.27412

**Published:** 2022-07-28

**Authors:** Fady Hatem, Hassan Baig, Foad Khaldas, James Lucocq

**Affiliations:** 1 General Surgery, Cairo University, Cairo, EGY; 2 Department of General Surgery, Queen Elizabeth University Hospital, Glasgow, GBR; 3 Department of General Surgery, Princess Alexandra Hospital, Harlow, GBR; 4 Department of General Surgery, Victoria Hospital, Kirkcaldy, GBR

**Keywords:** laparoscopy, predictors of negative appendicectomy, emergency appendicectomy, outcome of appendicectomy, negative appendicectomy rate

## Abstract

Introduction

A negative appendicectomy rate (NAR) is defined as the portion of pathologically normal appendices removed surgically in patients suspected of having acute appendicitis. The lifetime risk of acute appendicitis is 8.6% for males and 6.7% for females; contrarily, the lifetime risk of appendicectomy is 12% for males and 23.1% for females. This study aims primarily to evaluate the true NAR in females of childbearing age to offer insight into potential strategies to reduce the number of unnecessary operative procedures carried out, along with their associated morbidity and mortality.

Methods

All emergency appendicectomies over a one-year period were retrospectively identified and collected from a single tertiary care centre. Preoperative clinical, laboratory and postoperative histopathological data were collected. The negative appendicectomy rate in subgroups divided by biomarkers and radiological imaging findings were analysed. The diagnostic value of these modalities in the context of acute appendicitis was found by calculating the sensitivity, specificity, positive predictive values, and negative predictive values.

Results

A total of 417 patients were included (median age 26; M:F, 0.7:1.0). The overall negative appendicectomy rate was 35.0% (146/417). Two-hundred sixty-one patients underwent an appendicectomy in the child-bearing age group. The NAR was significantly higher in those females with raised WBC and C-reactive protein (CRP) compared to their male counterparts (p-value -<0.001).

Conclusion

Women of childbearing age have a higher NAR of 43% when compared to the general population of 35%. Preoperative tests, including ultrasound scans, computed tomography and inflammatory markers in blood tests, help direct those who would benefit from surgery to the operating theatre, however, no test alone is suitably sensitive or specific. To reduce the NAR, management options include a return to observation and serial examination, increased use of low-dose CT or a commitment to improving the performance of ultrasonography.

## Introduction

Acute appendicitis remains among the most common abdominal emergencies worldwide [1]. The American Journal of Epidemiology reports a lifetime risk of acute appendicitis at 8.6% for males and 6.7% for females; contrarily, the lifetime risk of appendicectomy is 12% for males and 23.1% for females. Its cause remains poorly understood, with few advances made in recent years. Obtaining a confident preoperative diagnosis still proves a challenge due to a multitude of differential diagnoses with similar presentations, as the possibility of appendicitis must be investigated in any patient presenting with an acute abdomen [2]. Biomarkers and imaging are valuable adjuncts to history and examination; however, their limitations mean that clinical assessment remains the mainstay of diagnosis [3].

Diagnostic accuracy achieved by history and physical examination has been reported at about 80% overall for both men and women. For males specifically, this diagnostic accuracy has been reported to range from 78% to 92%; however, for females, it is reported at just 58% to 85% [4].

A negative appendicectomy rate (NAR) is defined as the portion of pathologically normal appendices removed surgically in patients suspected of having acute appendicitis [5]. Although there is no widely accepted NAR to compare outcomes against, recent British studies have revealed NARs ranging from 19% to 33.9%. A summarised study collating 11 large international studies reported an overall NAR of 26% [6].

Recently, radiological imaging techniques, such as ultrasound scans (USS), computed tomography (CT), and magnetic resonance imaging (MRI), were evaluated as diagnostic modalities in acute appendicitis and have been shown to improve diagnostic accuracy and patient outcomes [7]. However, the routine use of imaging studies in all patients is not well established, and there is much variation between published data regarding the accuracy of these modalities and practical findings. USS imaging tends to be used primarily for women of childbearing age due to its lack of radiation exposure as well as being faster to carry out as compared with other imaging modalities. Despite USS being frequently used, many of these are reported with the appendix not being visualized, and therefore, not adding to the patient's clinical management.

This study aims primarily to evaluate the true NAR in females of childbearing age to offer insight into potential strategies to reduce the number of unnecessary operative procedures carried out, with their associated morbidity and mortality. Females of childbearing age are a higher risk group of patients due to the potential risk to reproductive organs and/or products of conception.

Second, this study aims to examine the value of biomarkers and imaging modalities in the diagnosis of acute appendicitis in daily practice, to form clear guidance on the management of acute appendicitis to optimize the use of resources and improve patient outcomes, particularly in the focus patient group.

## Materials and methods

All emergency appendicectomies over a one-year period were collected and retrospectively identified from a single tertiary care centre. This was subsequently limited to include both male and female patients between 16 and 45 years of age. Preoperative clinical, laboratory and postoperative histopathological data were collected. Preoperative imaging was determined by interrogating the Picture Archiving and Communication System (PACS). Appendiceal histology was reported by a team of histopathologists. Any degree of inflammation (acute, subacute or chronic or malignancy (e.g. carcinoid)) leading to a histopathological diagnosis of appendicitis was considered true appendicitis for this study and thus was not included as a negative appendicectomy. Anything outwith this pathology was considered a negative appendicectomy for the purposes of this study.

Preoperative biomarkers taken from the most recent blood tests prior to operating, particularly WBC and C-reactive protein (CRP) and radiological imaging (USS and CT of the abdomen and pelvis) findings were evaluated. A WBC cut-off of ≥10x10^9^/L and CRP cut-off of ≥5 mg/L were regarded as positive. The negative appendicectomy rate in subgroups divided by biomarkers and radiological imaging findings were analysed. The diagnostic value of these modalities in the context of acute appendicitis was found by calculating the sensitivity, specificity, positive predictive value, and negative predictive value. Patients under five years of age were excluded from this study.

The overall NAR in females between 16 and 45 years of age undergoing emergency appendicectomy was compared with males of the same age.

Results were analysed using STATA 17.1 software (StataCorp. 2021. Stata Statistical Software: Release 17). Comparative analysis was conducted using the chi-squared and Mann-Whitney U tests.

## Results

A total of 417 patients were included (median age 26; M:F, 172:245) (Table 1). The overall negative appendicectomy rate of the entire cohort was 35.0% (146/417). The diagnostic accuracies of inflammatory markers are demonstrated in Table 2. The group with the lowest positive predictive value (PPV) for appendicitis was the female group aged 16-45 (64%). In the male group, also of age 16-45, PPV was 87%. The combined diagnostic accuracy measures of patients with USS and CTAP (CT scan of the abdomen and pelvis) imaging are demonstrated in Table 3.

**Table 1:** Overall Data

Characteristics of Data	Results
Median age in years (range)	26 (5-84)
Male: Female	172:245
Raised white blood cells/C- reactive protein	291 (69.8)
Imaging	
Ultrasound scan	169 (40.5)
Computed tomography of abdomen and pelvis	143 (34.4)
Mode of Operation	
Laparoscopic	359 (86.1)
Laparoscopic converted to open	15 (3.6)
Open appendicectomy	43 (10.3)
Histopathological Findings	
Positive appendicectomy	
Acute inflammation	193 (46.3)
Subacute inflammation	43 (10.3)
Chronic inflammation	31 (7.4)
Malignancy (e.g. carcinoid)	4 (1.0)
Negative appendicectomy	
Normal	118 (28.3)
Hyperplasia	15 (3.6)
Fibrosis	5 (1.2)
Other	8 (1.9)

**Table 2:** Diagnostic accuracy measures of patients with raised white blood cells and C-reactive protein

	Sensitivity	Specificity	Positive predictive value	Negative predictive value	Likelihood positive	Likelihood negative
All patients	0.85	0.58	0.79	0.69	2.05	0.25
Female 5-15	0.77	0.86	0.87	0.75	5.50	0.27
Male 5-15	0.84	0.67	0.88	0.60	2.55	0.24
Female 16-45	0.79	0.54	0.64	0.72	1.72	0.39
Male 16-45	0.91	0.50	0.87	0.61	1.82	0.18
Female ≥46	0.97	0.50	0.92	0.75	1.94	0.06
Male ≥46	0.96	0.39	0.72	0.83	1.57	0.10

**Table 3:** Diagnostic accuracy measures of patients with an ultrasound scan and computed tomography of abdomen and pelvis (both males and females)

Imaging modality	Sensitivity	Specificity	Positive predictive value	Negative predictive value
Ultrasound scan	38.2	7.5	80.6	64.7
Computed tomography of abdomen and pelvis	90.0	63.6	82.5	52.1

Females of childbearing age

Two-hundred sixty-one patients underwent an appendicectomy in the childbearing age group (Table 4). The NAR was significantly higher in those females with raised WBC/CRP compared to their male counterparts (p-value 0.001). Comparisons of the USS and CT groups were limited by low numbers in the male gender group. The NAR in those with a non-visualised appendix on USS was significant (56.7%).

**Table 4:** NAR in Females and Males, 16-45 years WBC: white blood cells; CRP: C-reactive protein; NAR: negative appendicectomy rate

	NAR of patients aged 16-45 years (%)			
	All (n=261)	Females (n=156)	Males (n=105)	p-value
Histology				
Normal WBC/CRP	67.1 (55/82)	71.0 (44/62)	55.0 (11/20)	0.19
Raised WBC/CRP	24.6 (44/179)	35.1 (33/94)	11.8 (10/85)	<0.001
Ultrasound scan findings				
Inflamed	19.0 (4/21)	15.0 (3/20)	100.0 (1/1)	0.19
Appendix not visualized	54.8 (51/93)	56.7 (51/90)	33.3 (1/3)	0.42
Appendix not visualized (raised WBC/CRP)	38.0 (19/50)	38.3 (18/47)	33.3 (1/3)	0.86
Appendix not visualized (normal WBC/CRP)	76.7 (33/43)	76.7 (33/43)	N/A	-
Computed tomography of abdomen and pelvis findings				
Inflamed	86.4 (8/59)	78.6 (6/28)	6.5 (2/31)	0.09
Normal	60.0 (6/15	50.0 (4/8)	42.9 (3/7)	0.78

## Discussion

Overall, this study reveals that there is a high level of diagnostic uncertainty in females presenting with right iliac fossa pain. This study reveals that females of childbearing age have a significantly higher NAR at 43% as compared to the general population in this study, at 35%. Previous studies have demonstrated an overall NAR between 19% to 33.9%, which is a very high volume of patients given that acute appendicitis remains among the commonest acute surgical presentations. The importance of this study is its specific focus on women of childbearing age, as this population does not have clear evidence of having a higher negative appendicectomy rate as compared with males of the same age group. The main reasons for this finding are likely related to first, the imaging modalities used, and second, the differing anatomy of the sexes.

There are several methods through which further information could be ascertained as to why this specific population has a high NAR when compared with other patient populations. One of the methods that would have enhanced this study would be to reassess the patients who underwent a negative appendicectomy to evaluate their symptoms, or if any alternative diagnosis was reached for their pain if it still had not resolved. There have been studies reporting that women are at a higher risk of developing functional abdominal pain symptoms when compared with males [8].

This study revealed that the value of raised inflammatory markers can often be limited in the diagnosis of acute appendicitis due to their lack of specificity. However, normal inflammatory markers demonstrated a negative predictive value of acute appendicitis of 71%. This study focussed on white blood cell count and CRP as the inflammatory markers, as these have previously been identified as the most useful inflammatory markers of acute appendicitis [9]. This finding is contrary to some previous studies that stated no patients with acute appendicitis would have a normal CRP and white blood cell count [10]. Negative CRP has been shown to exclude appendiceal perforation, however, these results can only be suitable to guide management in conjunction with other investigations.

In two systematic reviews, ultrasonography has a composite sensitivity between 78 and 86% and specificity between 81 and 83 % for appendicitis [11-12]. Contrarily, however, our study displays a low sensitivity and specificity of ultrasonography in acute appendicitis. This was largely because only two patients who had a normal ultrasound were taken to the theatre (and both were subsequently found to have inflamed appendices). One of the problems associated with the use of USS imaging in acute appendicitis is that is largely operator and patient-dependent. USS imaging is of value if sufficient radiological expertise is available and is suitable for patient body habitus.

The alternative imaging modality, which is commonly used, is CT of the abdomen and pelvis. Although CT imaging has many benefits over USS imaging in the context of acute appendicitis, its risks must still be acknowledged. Some studies have reported a lifetime cancer risk of one in 250 women where oral or intravenous contrast was administered [13]. This risk remains the reason why this imaging is preferably avoided.

Apart from radiological imaging, diagnostic laparoscopy remains a common modality for confirming the diagnosis, however, it also has risks and benefits. In combination, the use of these three imperfect diagnostic techniques used in various combinations still results in diagnostic uncertainty and a high NAR. Interestingly, a recent survey put diagnostic laparoscopy as the investigation of choice for investigating suspected appendicectomy, especially in young females [14]. This approach may further increase the NAR but still result in shorter admissions and lower morbidity overall.

This study had multiple limiting factors, which, if addressed, would significantly add to the impact of this study. First, the sample size can be enlarged through multi-centre data collection; and second, carrying this study out prospectively would enhance the data collection and sample size

This study is limited to being a retrospective analysis of the histopathological samples. Therefore, the number of appendicectomies avoided due to negative scans and/or blood tests or alternative diagnoses managed without operative intervention were not explored. However, we found that there is a great discrepancy between published results and our practical findings, especially in the imaging modalities utilised.

## Conclusions

This study reveals that females of childbearing age have a significantly higher NAR when compared to the general population. Very little data are present highlighting the NAR in this particular patient population. Preoperative tests including USS, CT and inflammatory markers in blood tests help direct those who would benefit from surgery to the operating theatre; yet no test alone is suitably sensitive or specific. They are used in combination and with clinical judgement, however, this still results in a 43% NAR in this particular population. To investigate further, eventual diagnoses of these women with negative appendicectomies can be sought; however, diagnosing appendicitis preoperatively in females of childbearing age remains a difficult challenge. The traditional preference for ultrasound scanning in the UK compared to CTAP in young populations is primarily due to the potential hazards of irradiation, but USS commonly does not visualise the appendix in practice and has low sensitivity and specificity for appendicitis, however, after positive USS, the NAR dropped to 15%.

This study shows that female patients with raised inflammatory markers are less likely to have a positive appendicectomy compared to their male counterparts likely due to alternative pathology. To reduce the NAR, management options include a return to observation and serial examination, increased use of low-dose CT or a commitment to improving the performance of ultrasonography.

## References

[1] Di Saverio S, Podda M, De Simone B (2020). Diagnosis and treatment of acute appendicitis: 2020 update of the WSES Jerusalem guidelines. World J Emerg Surg.

[2] Addiss DG, Shaffer N, Fowler BS, Tauxe RV (1990). The epidemiology of appendicitis and appendectomy in the United States. Am J Epidemiol.

[3] Bhangu A, Søreide K, Di Saverio S, Assarsson JH, Drake FT (2015). Acute appendicitis: modern understanding of pathogenesis, diagnosis, and management. Lancet.

[4] Drake FT, Florence MG, Johnson MG (2012). Progress in the diagnosis of appendicitis: a report from Washington State's Surgical Care and Outcomes Assessment Program. Ann Surg.

[5] Raja AS, Wright C, Sodickson AD (2010). Negative appendectomy rate in the era of CT: an 18-year perspective. Radiology.

[6] Lim J, Pang Q, Alexander R (2016). One year negative appendicectomy rates at a district general hospital: a retrospective cohort study. Int J Surg.

[7] Mostbeck G, Adam EJ, Nielsen MB (2016). How to diagnose acute appendicitis: ultrasound first. Insights Imaging.

[8] Drossman DA, Li Z, Andruzzi E (1993). U.S. householder survey of functional gastrointestinal disorders. Prevalence, sociodemography, and health impact. Dig Dis Sci.

[9] Kaya B, Sana B, Eris C, Karabulut K, Bat O, Kutanis R (2012). The diagnostic value of D-dimer, procalcitonin and CRP in acute appendicitis. Int J Med Sci.

[10] Grönroos JM, Grönroos P (1999). Leucocyte count and C-reactive protein in the diagnosis of acute appendicitis. Br J Surg.

[11] Terasawa T, Blackmore CC, Bent S, Kohlwes RJ (2004). Systematic review: computed tomography and ultrasonography to detect acute appendicitis in adults and adolescents. Ann Intern Med.

[12] van Randen A, Bipat S, Zwinderman AH, Ubbink DT, Stoker J, Boermeester MA (2008). Acute appendicitis: meta-analysis of diagnostic performance of CT and graded compression US related to prevalence of disease. Radiology.

[13] Smith-Bindman R, Lipson J, Marcus R (2009). Radiation dose associated with common computed tomography examinations and the associated lifetime attributable risk of cancer. Arch Intern Med.

[14] Jaunoo SS, Hale AL, Masters JP, Jaunoo SR (2012). An international survey of opinion regarding investigation of possible appendicitis and laparoscopic management of a macroscopically normal appendix. Ann R Coll Surg Engl.

